# Service delivery in Kenyan district hospitals – what can we learn from literature on mid-level managers?

**DOI:** 10.1186/1478-4491-11-10

**Published:** 2013-02-26

**Authors:** Jacinta Nzinga, Lairumbi Mbaabu, Mike English

**Affiliations:** 1 Health services and Research Group, Kenya Medical Research Institute/Wellcome Trust Research Programme, Nairobi 00100, PO Box 43640, Nairobi, Kenya; 2 Nuffield Department of Medicine & Department of Paediatrics, University of Oxford, Oxford, UK

**Keywords:** Competencies, Health care settings, Hospitals, Middle managers, Mid-level managers, Roles

## Abstract

**Background:**

There is a growing emphasis on the need to tackle inadequate human resources for health (HRH) as an essential part of strengthening health systems; but the focus is mostly on macro-level issues, such as training, recruitment, skill mix and distribution. Few attempts have been made to understand the capability of health workers, their motivation and other structural and organizational aspects of systems that influence workforce performance. We have examined literature on the roles of mid-level managers to help us understand how they might influence service delivery quality in Kenyan hospitals. In the Kenyan hospital settings, these are roles that head of departments who are also clinical or nursing service providers might play.

**Methods:**

A computerized search strategy was run in *Pub Med*, *Cochrane Library*, *Directory of Open Access Journals Social Science Research Network*, *Eldis*, *Google Scholar* and *Human Resources for Health* web site databases using both free-text and MeSH terms from 1980 to 2011. In addition, citation searching from excluded and included articles was used and relevant unpublished literature systematically identified.

**Results and discussion:**

A total of 23 articles were finally included in the review from over 7000 titles and abstracts initially identified. The most widely documented roles of mid-level managers were decision-making or problem-solving, strategist or negotiator and communicator. Others included being a therapist or motivator, goal setting or articulation and mentoring or coaching. In addition to these roles, we identified important personal attributes of a good manager, which included interpersonal skills, delegation and accountability, and honesty. The majority of studies included in the review concerned the roles that mid-level managers are expected to play in times of organizational change.

**Conclusion:**

This review highlights the possible significance of mid-level managers in achieving delivery of high-quality services in Kenyan public hospitals and strongly suggests that approaches to strengthen this level of management will be valuable. The findings from this review should also help inform empirical studies of the roles of mid-level managers in these settings.

## Background and context

We face major challenges in providing evidence-informed health care; the right interventions to the right people at the right time in routine settings. This challenge is particularly acute in low-income settings [1,2] and we, and others, have described specific major failures in hospital care in Africa [3-5]. The provision of poor-quality care has often been attributed to inadequate knowledge and skills compounded by broader system failures and low staff numbers. The need to tackle inadequate human resources for health (HRH), as an essential part of strengthening health systems was emphasized in the 2006 World Health Report [6]. However, the focus of attention has to date largely been on macro-level issues related to workforce training, recruitment, retention, skill mix and distribution. More recently, attention has turned to the capability of health workers, their motivation and other structural and organizational aspects of systems that influence workforce performance [6,7]. As we make efforts to expand access to a skilled workforce to improve delivery of key interventions we also need to better understand, articulate and develop roles within the health workforce that support effective service delivery.

District hospitals in low-income African settings often have between 60 and 300 inpatient beds and similar numbers of total staff [8]. These numbers, although small by developed-country hospital standards, are typically organized as multiple service delivery units. These reflect the nature of care (outpatient and inpatient) and service type (for example, adult surgical or paediatric wards). Traditionally, the focus in low-income settings among those expected to lead such units has been on technical competence, yet it is increasingly recognized that leadership, supervision, information dissemination and communication are major mediators and moderators of the quality and effectiveness of health care [9,10]. Furthermore, in previous work we identified such unit heads as a key influence on the effectiveness of an intervention aimed at promoting uptake of recommended practices [11]. We therefore set out to explore what roles such units heads need to play that go beyond their technical competence and that help promote uptake of effective interventions and delivery of high-quality services. We therefore carried out a literature review with a particular focus on empirical literature on mid-level managers in hospitals. The specific aim was to characterize important, non-technical roles of relevance to service delivery unit heads in Kenyan hospitals. We briefly provide a broader introduction to Kenya’s health sector before presenting our findings. 

### Policy context in Kenya

Kenya’s evolving health policy context has much in common with that in many Anglophone African countries. The late 1980s saw the adoption of measures inspired mainly by the New Public Management rhetoric [12], such as the introduction of performance management and advocacy for the “empowerment” of managers. In 1992, in the public sector, District Health Management Boards (DHMBs) were created in the country’s 71 districts. In theory at least these were responsible for: collaboration and coordination with other district-level health sector actors; planning and regulation of district health systems; and resource generation through the capacity to set user charges [13]. The administrative roles of these boards were endorsed in subsequent National Health Sector Strategic Plans (for the periods 1999–2004 and 2005–2010) while the most recent policy initiatives [14], including those espoused in Kenya’s new constitution [15], suggest a continued devolution of powers to senior managers in new county administrations and their hospitals. Alongside this shift there has been an increasing discourse in policy on the need for management skills, if not for professional managers, but over the last twenty years this discourse has largely focused on senior management.

**Figure 1:**
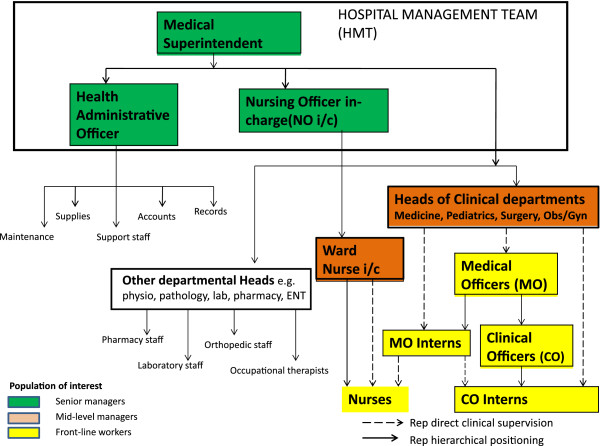
Generic organogram of district hospitals in Kenya. *Medical officers here refer to physicians while clinical officers are “non-physician” clinicians as described in [16].

Within the hospital setting, the senior management is made up of a hospital management team that holds administrative power. This comprises persons in charge of administration, nursing, pharmacy and allied health services and is typically led by the medical superintendent. Those in charge of different clinical service units or departments are invariably clinicians and nurses who operate without any specific departmental administrators [3]. They are expected to plan and advocate for resources, although they are unlikely to have direct control over a specific departmental budget. Such individuals also supervise teams of front-line workers, either medical or nursing, and contribute directly to service delivery. The lead clinician may have a higher degree in an appropriate medical specialty or, especially in smaller rural hospitals, may still have a general medical qualification. Specialist doctors in leadership roles may have as few as 5 years’ total work experience (including their 3 years training), although some will have many more. General medical practitioners in smaller hospitals may have only 1 year of work experience before taking charge of a department. The nurses leading departments tend to have more work experience although very few at this level have any higher training in a specific clinical specialty (for example paediatric or surgical nursing). It is such personnel that are the focus of our concern (Figure 1).

Our prior experience is that the roles of such personnel go beyond direct service provision, and are poorly described in Kenyan policy and little discussed in the literature. Instead, what attention is given to hospital management tends to focus on the administrative roles of the hospital senior management teams. This research suggests that senior managers are often not well prepared [17,18] for this role although there are current efforts to build skills in these areas through “management training”. The potentially important management roles of those at the middle level of management are less well addressed.

## Methods

We set out to review the evidence on the clinical leadership and management roles of departmental and mid-level managers in hospital care. A combination of both free-text and MeSH terms, as described in Table 1, was used. The databases that were searched included: *Pub Med*, *Cochrane Library*, *DOAJ* (*Directory of Open Access Journals*), *SSRN* (*Social Science Research Network*), *Eldis*, *Google Scholar* and *HRH* (*Human Resources for Health* web site). In addition, we searched the bibliographies of retrieved manuscripts and sought out grey literature, including conference reports and editorial letters.

**Table 1:** Search terms

**Target population (combined with** “**OR”)**	**Role category (combined with** “**OR”)**	**Setting (combined with** “**OR”)**
Clinic* OR	Roles OR	Health OR
Lead* OR	Competencies OR	Hospital OR
Manag* OR	Qualities OR	Healthcare OR
Management (MeSH) OR	Responsibilities OR	Health sector OR
Clinician manager OR	Strategies OR	Health organization OR
Clinician leader OR	Challenges OR	Human Resource Leadership OR
Physician manager OR	Barriers OR	Healthcare Systems OR
Physician leader OR	Facilitators OR	Health Services
Mid-level manager OR	Behaviors OR	
Middle manager OR	Style	
Departmental leader OR		
Departmental manager		

**Figure 2:**
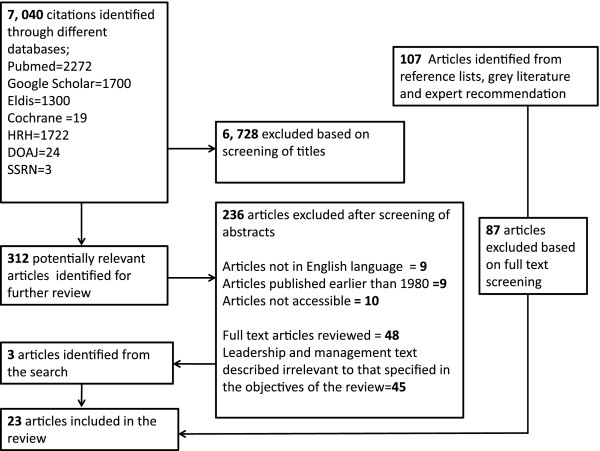
Exclusion criteria.

Articles published in English after 1980, irrespective of the study design, were considered for inclusion if the study referred to hospitals or included mid-level managers; reported mid-level managers’ roles in practice; or reported the impact, perceptions of or any other outcomes of these roles. Articles for inclusion were identified by the main author (JN) after discussion with a second author (ME) in cases of uncertainty. The search strategy yielded 7040 citations in total from bibliographic databases and 107 articles from our search of the grey literature and expert recommendation. After applying pre-specified exclusion criteria (see Figure 2), 23 articles were available for inclusion in our review ([11,19-40], see Additional file 1). These studies were conducted between 1980 and 2011 and all reports were from developed countries. Nine studies were exclusively conducted in the UK, five studies in the USA, two in Canada, one in Australia and six did not specify the location. Most were case studies (*n* = 10), but other designs reported included surveys (*n* = 2) and social network analysis (*n* = 1). Four reports did not clearly specify the methodological design. Also included were three reports categorized as correspondence, essays, or opinion pieces. Studies mostly focused on nurse managers. One study focused on a variety of industries that included the health service industry.

A standard data abstraction table was used to document the identified literature systematically in terms of the nature of the study, its location, research question and the key findings. Tabulated summaries were used to highlight key findings of relevance to our question: what roles ideal mid-level managers might play in ensuring delivery of high-quality clinical services. We considered a role to be “an organized set of behaviours linked to a particular position in an organization”, as defined by Guo and Calderon [27]. As identified roles appeared, these were listed. Similar roles were then aggregated into broader thematic roles by referring to the original full texts in an iterative process until conceptually distinct role types were defined. Those articles providing insights on the specific role types identified were then re-examined in detail to help characterize this role type (see Additional file 1). We did not come across any study, of any design, that attempted to intervene specifically at the mid-level manager (MLM) level. While it was not a formal aim of this review to determine whether better hospital services are linked to better mid-level management, we did take note of the authors’ observations or conclusions in this area.

## Results and discussion

There appeared to be no agreed definition of an MLM. For the purposes of this review, we adopt a broad definition of an MLM in a hospital as a manager who is directly involved in planning and coordinating the production of services that are specific to their own units, bridging the gap between senior-level management and front-line workers. Definitions within the reviewed studies lean more towards those managers who hold primary administrative responsibilities of heading department or units, as well as being in charge of clinical services, closer to clinical hybrid managers [40,41]. In many cases, however, the managers under study had no personal role in service provision.

**Figure 3:**
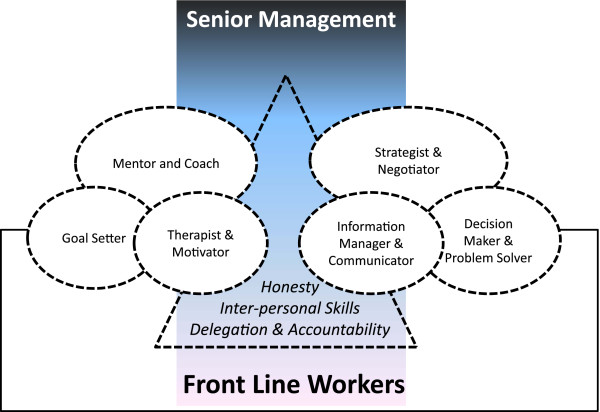
**Key roles and characteristics of effective MLMs in hospitals.** MLMs are shown (features encompassed in broken lines) as the interface between senior management, represented by the vertical rectangle (who act largely through MLMs with relatively little interface with front-line workers) and the front-line workers, represented by the horizontal rectangle.

Based on the identified literature, we now present the scope and nature of MLMs’ behaviours and roles that are reported to support achievement of organizational goals and delivery of high-quality hospital services. These roles are grouped into two main categories with examples of such roles or related personal attributes used to illustrate these categories. We identified six roles related to behaviour as expected actions and three personal attributes or attitudes that support the management role. The former category includes such roles as: mentor and coach, linked to roles as goal setter and motivator and therapist, and strategist and negotiator, linked to roles as information manager and decision-maker and problem-solver. The latter category includes personal attributes or attitudes that promote effectiveness, good interpersonal skills delegation and accountability, and honesty. The themes are described in more detail and summarized in Figure 3.

### Mentor and coach

A middle manager’s role as a mentor and a coach ideally involves guiding staff to advance their knowledge and skills through continuous learning. Excellent MLMs, therefore, must make an effort to keep themselves updated, enabling them to coach others, as discussed by Nilson [19] and Pappas *et al*. [31]. Additionally, Guo and Calderon [27] explain that such a role helps create the informal social underpinning of the social networks through which MLM can build consensus and then direct and promote change to improve departments and organizations. This role also sees MLMs as professional role models and allows them to set and articulate goals and act as therapists and motivators.

### Goal setting and goal articulation

Roles in this domain are summarized by Currie [24], who explains that MLMs must translate higher-level goals (corporate strategy) into contextually relevant local goals and action plans, individualized objectives and short-term operational foci of behaviour. Balding [20] and Patrick and Laschinger [42] explain that this process should result in the creation of a sense of cohesion around local goals, aligned with whole organizational goals, by building supportive coalitions while providing a work (micro-) environment that supports goal achievement [19,20,27,42]. This role obviously draws on other competencies, such as communication (clarifying and articulating goals for the intended audience) and motivation, and is well described in Balding’s work on MLMs’ role in quality improvement in Australian hospitals [20].

### Therapist and motivator

Within the broad arena of relations behaviour, MLMs are required to take on pro-active supporting roles, especially during periods of profound change. Embertson [26] and Huy [28] explain that MLMs attend to employees’ emotional well-being in times of change by monitoring and controlling elements of the system so that employees are satisfied and work demands are met. Balogun [21] elucidates that this role involves first undertaking personal change so that MLMs interpret the implications of the change for themselves in terms both of the way they think and of how they carry out their work. This informs all their other roles, allows them to provide support and direction, and is in fact the key task of MLMs [21]. Thus, the MLMs can emerge as change agents who absorb and diffuse best practices, providing opinion leadership, reassurance and support to those who need to change, as shown by Dopson and Fitzgerald [25]. Such a role is critical, as while change may be planned by top-level management the official discourse is typically unable to address employees’ emotional well-being. Indeed, an effective MLM can “facilitate adaptability” and even produce more radical changes than expected by the executive management [24].

### Strategist and negotiator

Bolton reports that MLMs are commonly reported as actively undertaking strategic changes, acting as representatives of new ideas or of championing changes in their units [22]. In a project to implement quality improvement, Balding reports that MLMs were responsible for significant improvements in perceptions of this initiative across the organization [42]. The important role of the MLM in negotiating improved implementation of quality improvement was also seen in other settings, where it led to a greater appreciation of the importance of MLMs in achieving strategic objectives [23]. Demonstrating that MLMs may not just be mediators but initiators, Currie found that, in a hospital trust where business managers were promoting marketing concepts to improve performance, MLMs were able to take the lead in building relationships with “customers” for their service area [43].

The ability of MLMs to embrace roles as strategist and negotiator requires MLMs to understand a new or existing strategy and build consensus around this strategy. However, according to Balogun [21], Currie [43] and Noorein Inamdar *et al*. [44], this is only likely if MLMs feel it is in the best interest of the hospital. The work of [21], Currie [43] and Noorein Inamdar *et al*. [44] also indicates that consensus building is a deeply embedded, socially constructed process and that the inclusion of middle-management teams in the strategy-development process, to which they can bring ideas based on their detailed local knowledge, can also reap positive rewards [30]. This overarching role provides the basis for linked specific roles as decision-maker and problem-solver, and as information manager and communicator.

### Decision-maker and problem-solver

Many studies, including those by Patrick and Laschinger [42] and Bolton [22], reported MLMs as being involved in making decisions and solving problems in the service areas where they were assigned [22,42]. McLarty and McCartney [29] report that discretionary decision-making is common at the point of care, on matters that impact day-to-day operations and that help resolve conflict within and among teams and levels of hospital authority. According to Nilson [19] and Patrick and Laschinger [42], excellent MLMs are those who, when making decisions, focus on options that are best for the people in their organizations, demonstrating a sense of responsibility to their staff, patients and superiors [19,28,42]. Huy [28] and Timmreck [34] highlight that MLMs also demonstrate entrepreneurship when solving problems; because they are close to day-to-day operations, they know better than anyone where the problems are; however, they are also far enough away from front-line work that they can see new possibilities for solving problems [28,34]. Embertson [26] and Balogun [21] point out that a particularly important arena in which MLMs make decisions and solve problems is when organizations are transitioning through major changes. Here, MLMs are called upon to “keep business going”, a role that requires juggling priorities to balance the pace between continuity and change [21,26,28].

### Information manager and communicator

Classical managerial functions of MLMs in health services include the provision of effective oral and written communication. Indeed McLarty and McCartney [29] and Timmreck [34] report that the time taken up in direct communication with others, including patients, staff, and hospital management, uses up the greatest proportion of a manager’s time. Checkland *et al*. [40] and Wallace and Corey [36] describe that, linked with ideas of MLM as strategists and negotiators, are requirements of MLMs to act not just as conduits for information flow but as actors able to synthesize, package and use information to communicate with diverse groups and effectively bring about change [36,40]. In addition to framing information for transmission from higher levels in the system to lower levels, this role in synthesis and management can be used in reverse to influence senior managers. Checkland *et al*. [40] and Currie [24] report such examples as restructuring decisions about what programmes to close, whom to layoff, and where to cut costs [40,44] or championing alternative approaches to current management strategy [24,40].

This role requires MLMs to package information in a way that makes sense to the recipients without losing the essence of the intend message. However, a coherent message will only be of value if disseminated and, as Checkland *et al*. [40], Huy [28] and Pappas and Wooldridge [30] explain, MLMs may have access to powerful, informal, social networks in the organization because of their strategic positioning in the hierarchy, as well as to more formal channels of communication [28,30,40]. Such networks are typically based on unwritten obligations and favours that are traded, giving effective middle managers a significant amount of informal leverage. Indeed, MLMs understand and can use this informal power to produce “knowledge centres” by working across organizational networks and creating an environment that encourages the sharing of information, as discussed by Embertson [26]. According to Fitzgerald *et al*. [38], MLMs can thus promote or impede a change initiative: as a result it has proven important to pay attention to the social structure and the flow of information within an organization.

To allow MLMs to play, and be effective in, the key roles outlined above they also need to behave in ways that enable their acceptance by front-line workers and senior managers and that allow them to effectively bridge these different hierarchical levels.

### Interpersonal skills

Nilson [19] argues that for MLMs to be effective, research suggests that they should be empathetic, emotionally intelligent, approachable and have the ability to put people at ease with a rapport that invites people to come to them with problems, questions and suggestions. These skills, according to Dopson and Fitzgerald [25] and Wallace and Corey [36], play crucial roles in developing and maintaining the quality of relationships that will be needed for effective, coordinated group behaviour. These affect-based relationships are characterized by loyalty, trust, respect and transparency and by the avoidance of demeaning and abusive behaviour. Checkland *et al*. [40], Pappas *et al*. [31] and Timmreck [34] add that these relationships provide a platform for high expectations of employee behaviour and are felt to enhance work performance.

### Honesty

In describing middle managers in health-care organizations, Nilson [19] states that excellent MLMs hold high standards in developing relationships with others and are always honest, keeping promises and having no hidden agenda. Noorein Inamdar *et al*. [44] add that this notion of honesty extends to matching words with actions. Middle managers must earn trust by being forthright, hard-working, and delivering on their commitments, thereby gaining credibility and being able to exert agency in their areas of responsibility. Honesty in this broad sense (akin to trustworthiness), therefore, helps cement good relationships between senior managers and MLMs and between MLMs and other professionals, promoting collegiality that forms a foundation for service improvement, as reported by Fitzgerald *et al*. [38].

### Delegation and accountability

In an effort to encourage distributed leadership, middle managers should share responsibility and accountability with the staff they are responsible for, as explained by Huy [28]. Nilson [19] adds that good MLMs hold themselves accountable and hold staff under their supervision to account for their actions. In addition to this, Timmreck [34], in describing the classical functions of MLMs in health services, states that properly trained MLMs delegate both responsibility and authority clearly as a necessary part of promoting accountability.

### Summary of the literature

We encountered challenges in retrieving some publications, largely because of difficulty in accessing print-only articles not available in Kenya that attempts to contact authors directly did not overcome. Despite this, we feel the literature included was informative. In this review, literature characterizing MLM roles was limited by absence of data evaluating roles or identified tasks. Nonetheless, the articles we found provide a useful insight into factors that may indeed increase MLM influence on the quality of care delivered. These findings reveal that important factors, such as decision-making and problem-solving, championing change, communication and information synthesis, are basic roles and tasks for MLMs. Studies included in this review were commonly concerned with the roles MLM are expected to play in times of organizational change. These support a major role for MLMs as potential change agents and opinion leaders, who can either facilitate or impede efforts to improve services [22,25,38,45] because of their boundary-spanning position [23,24,46]. Middle-level managers may be better disposed to support change when they understand the proposed initiatives for improvement and their role in it, if it is in line with their own values and fits the context in which they work, and if they feel supported by senior management [23,47,48]. Our findings are broadly consistent with those of Birken *et al*. [49], who focused on MLMs’ roles as agents of translation of policy into action, condensing these roles into those of diffusion, synthesis, mediation and marketing. Drawing on reports of empirical work, we preferred to maintain a greater level of granularity in our summary of the roles that MLMs who are also clinical or nursing service providers might play in a Kenyan hospital setting. We also highlight that these roles allow influence and information flow to be bidirectional and that at times MLMs may be aligned with and act with either senior managers or front-line workers. Given their apparent pivotal role and the radical changes needed within low-income country health systems, it is perhaps surprising that MLMs have been so ignored in these settings.

In addition to the roles or actions that MLMs must take to promote effective service delivery, it is important to recognize the value of how MLMs create and maintain important social relations. Thus, MLMs should be able to build and draw on social networks comprising affect-based linkages and to do this they must embrace their role as managers and engender loyalty, trust and respect, and be considered honest and straightforward. Yet this area is relatively understudied [22,50-52]. Perhaps this is because most leadership and management studies are directed at the business world, where the focus is on profits, unlike health care, which concerns itself with people-centred outcomes [27,53,54].

## Relevance to Kenyan hospitals

Previous work in Kenyan public hospitals has revealed leadership gaps and poor communication between senior administration and lower cadres as an impediment to achieving better practice [55,56]. Management training for senior health professionals has been recognized as a priority and is now being provided [57]. This review highlights some of the roles that MLMs can play towards achieving delivery of high-quality services in public hospitals in places like Kenya, even though the literature reviewed was from high-income settings. This relevance is based on a number of parallels across contexts. Clinical MLMs in both settings often have a significant professional identity with considerable autonomy within their work and organizational setting. Such autonomy increasingly results in calls for greater accountability, with MLMs having to accept greater levels of responsibility for management. Prompting clinicians to accept management roles is the fear of losing authority and of being treated as simply technicians. Arguably, this results in the emergence of the hybrid clinical manager across many settings. This is likely to be particularly true as Kenya undergoes both major administrative changes resulting from greater devolution [15] and because, like other low-income settings, major changes are needed to improve service delivery now and to promote continuous organizational learning and improvement. To achieve this, better support for health professionals who are also MLMs is required in addition to that focusing on the role played by senior managers in Kenya (see Table 2). Such training should emphasize the development of personal attributes that facilitate this role and may increase job satisfaction and performance [21,28,42].

**Table 2:** **Current efforts to address the management**/**leadership needs in Kenya**

**Leadership and management initiatives**	**Key actors**	**Target**
Approval for creation of additional 400 human resource management positions	Ministry of State for Public Service and awaiting budgetary allocation for implementation	Spread out to all levels of the health system in 2009
Review of top management positions, identifying key competencies, and gaps and providing recommendations for a way forward	Ministry of Health with assistance from Capacity Kenya Project	Launched in 2009 for health workers in public, private and faith based primary health care facilities
One month leadership development and management training programme	Leadership, Management and Sustainability Program/Management Sciences for Health	Launched in 2010 in 10 provincial hospitals in Kenya
A 5 day modular programme on leadership development and management in Coast and Nyanza province	Japan International Cooperation Agency	In 2009, aimed at strengthening the capacity and functions of the provincial and district health management teams
Facilitating the implementation of a 5 day leadership and management development programme in Nyanza Province	International Training & Education Center for Health, Family AIDS Care and Education Services (FACES) and the Provincial Health Management Team	Launched in 2009 to increase the skills and capacities of health program managers–including those on the FACES, provincial, and district health management teams
The Management Basics for Effective Health, a management course for District Health Management Team members	The German Technical Cooperation	Launched in 2010 in four district hospitals: Bondo, Butere/Mumias, Gucha and Vihiga
International Leadership Training (ILT-Africa) in hospital management for health workers with diverse professional backgrounds in both public and private sector	Inwent, Capacity Building International (Germany),	Launched in 2005 in Cameroon, Rwanda, Malawi, Tanzania and Kenya and ongoing Kenyan participants included nurses, health administrators and medical superintendents from district hospitals

## Conclusion

There is little research on MLMs in low-income settings, yet they appear to hold positions of considerable importance to those concerned with implementing better health services. The findings from this review should help inform empirical work whose focus is on examining such roles and the influence of clinical hybrid managers on care in public hospitals in Kenya. Of particular importance will be uncovering the professional power and autonomy that these managers hold in influencing their peers and followers, particularly in settings expected to change or improve their service delivery. Ideally such research might attempt to explore the implicitly proposed causal linkages between effective MLMs and hospitals’ performance in providing high-quality services.

## Abbreviations

DHMB: District Health Management Boards; DOAJ: Directory of Open Access Journal; FACES: Family AIDS Care and Education Services; HRH: human resources for health; ILT-Africa: International Leadership Training; MeSH: Medical Subject Headings; MLM: mid-level manager; SSRN: Social Science Research Network.

## Competing interests

The authors declare that they have no competing interests.

## Authors’ contributions

JN conceived of the idea for the manuscript, searched the literature, abstracted the data and conducted the analyses before preparing a first draft. ME critically reviewed the articles included in the review and contributed to the analytical interpretation of the data in discussion with JN. LM and ME provided inputs to the preparation of the draft. All authors read and approved the final manuscript.

## Supplementary Material

Additional file 1 Main findings from the studies included in the review. Click here for file
